# Cardiorespiratory mechanisms triggered during music perception in preterm infants and adults

**DOI:** 10.1016/j.isci.2025.113524

**Published:** 2025-09-11

**Authors:** Laura Lavezzo, Francisca Barcos-Munoz, Damien Benis, Juliette Margaria, Didier Grandjean, Enzo Pasquale Scilingo, Andreas Vollenweider, Stéphane Henriot, Mimma Nardelli, Petra Hüppi, Manuela Filippa

**Affiliations:** 1 Division of Development and Growth, Child and Adolescent Department, University of Geneva, Geneva, Switzerland; 2 Bioengineering and Robotics Research Centre E. Piaggio and Department of Information Engineering, University of Pisa, 56122 Pisa, Italy; 3 Neonatal Intensive Care Unit, University Hospital of Geneva, Geneva, Switzerland; 4 Swiss Center for Affective Sciences, Faculty of Psychology and Educational Sciences, University of Geneva, Geneva, Switzerland

**Keywords:** Physiology, Neuroscience, Developmental biology

## Abstract

Preterm newborns’ autonomic response to dynamic auditory stimuli is poorly understood. To examine how cardiac and respiratory systems adjust their rhythms in response to music, we assessed 18 preterm infants (gestational age 37.73 ± 0.80 weeks) and 19 adults across music listening. Heart rate dynamics, respiratory sinus arrhythmia (RSA), and cardiorespiratory coupling were analyzed. Studying the power spectral density of the interpolated interbeat interval series, both groups showed decreased high-frequency power during music listening. In preterm infants, RSA increased (*p* = 0.005), possibly suggesting a state of calm alertness, where the infant is physiologically prepared for interaction with environmental stimuli, while adults had decreased RSA (*p* = 0.003) and concomitant increased values of the low-frequency/high-frequency power ratio, possibly reflecting heightened alertness. While frequency domain cardiovascular responses to music were similar between preterms and adults, only the investigation of RSA and cardiorespiratory coupling measures revealed the delicate balance of autonomic dynamics in preterm newborns.

## Highlights

• Music increases RSA and cardiorespiratory coupling in preterm infants
• Music decreases HF power and RSA in adults
• Directional coupling patterns differ by age and condition
• Cardiorespiratory measures reveal subtle, age-specific autonomic balance

## Graphical abstract

**Figure:**
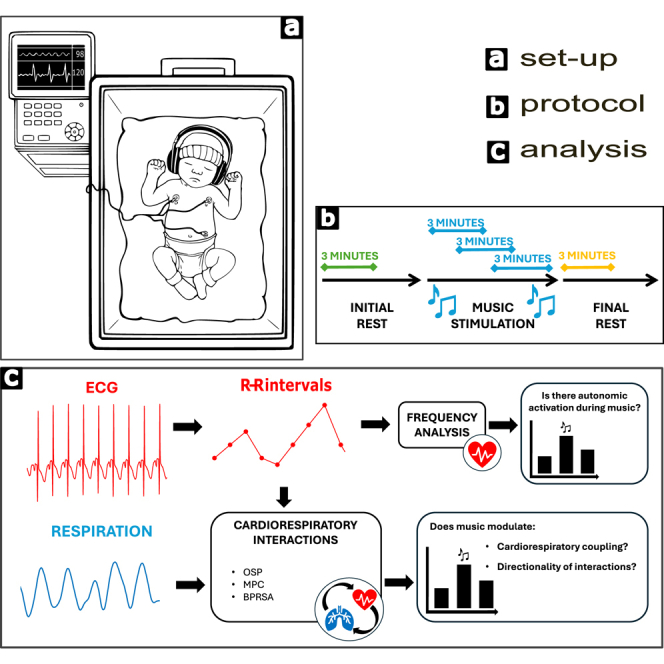

## Introduction

During hospitalization in the neonatal intensive care unit (NICU), preterm infants experience a phase of brain development in which activity-dependent plasticity is prominent before reaching term-equivalent age.^1^ During this period, they are exposed to many disturbing mechanical auditory stimuli, with significantly reduced exposure to human voices.^2^ This atypical auditory environment has increasingly been recognized to contribute to an increased risk of later adverse outcomes.^3^^,^^4^

Early enriching interventions, particularly music-based strategies, have shown potential to support neurodevelopment and autonomic regulation in this population,^5^^,^^6^ promoting autonomic stability and enhancing overall well-being in preterm infants.^7^^,^^8^^,^^9^ Recent studies also suggest that exposure to music and maternal singing can positively influence vital physiological functions, including heart rate, respiration, and stress regulation, by engaging the autonomic nervous system.^10^^,^^11^^,^^12^^,^^13^^,^^14^

Changes in cardiovascular dynamics, assessed through the study of interbeat interval (RR) series, reflecting the dynamic balance between sympathetic and parasympathetic activity, provided valuable insights into the infant’s capacity for self-regulation, especially while attending to external stimuli.^15^

Most studies investigating the effects of auditory stimulations on preterm infants’ cardiovascular system primarily focus on heart rate dynamics itself, often using high-frequency (HF) power as a marker of parasympathetic activity.^16^ While HF power is widely used, it does not directly quantify respiration-related influences on RR series because it relies on spectral approximations rather than explicitly separating respiratory and non-respiratory components.^17^ Traditional frequency-domain measures, although essential, do not clearly distinguish among the diverse pathways influencing the heart, limiting our understanding of which cardiorespiratory mechanisms are activated during, for example, music perception. This limitation is particularly relevant in preterm infants, whose autonomic and respiratory control systems are in a delicate balance during the early hospitalization period.^18^

Indeed, to better understand the mechanisms underlying responses to music in the preterm population, it is important to investigate the interaction between two principal physiological systems—the cardiac and respiratory systems—under neural regulation, which is conventionally recognized through respiratory sinus arrhythmia (RSA).^19^^,^^20^

RSA is a key physiological component reflecting the interplay between cardiac function and respiration. It is widely viewed as a non-invasive index of parasympathetic (vagal) cardiac control,^21^ but its interpretation should be cautious since RSA magnitude can be affected by multiple physiological and behavioral factors beyond vagal activity, including metabolic demands.^22^^,^^23^ In neonates and preterm infants, whose autonomic and respiratory systems are still maturing, these influences may vary considerably, potentially confounding straightforward interpretations of RSA as a pure marker of vagal function or interindividual differences.

RSA manifests as an increase in heart rate during inspiration and a decrease during expiration, making it a well-established marker of vagal tone and autonomic regulation. A stronger vagal influence is linked to a more pronounced coupling between the time series related to respiration and cardiovascular dynamics, resulting in elevated RSA levels. In adults, RSA serves as the predominant mechanism of cardiac modulation, where parasympathetic activity plays a crucial role, particularly in resting conditions.^24^^,^^25^ In preterm infants, however, RSA is more variable, reflecting ongoing maturation.^26^ Moreover, newborn respiratory rates often fall outside standard HF bands^17^^,^^27^ challenging conventional spectral analysis of interpolated RR (RRi) series. To address these issues, methods like orthogonal subspace decomposition have been used for robust estimation of RSA-related component in RRi series,^17^ which has been identified as a reliable marker of RSA,^28^ allowing clearer separation of respiratory versus other autonomic influences.

RSA has been widely used in infant studies as a physiological marker due to its non-invasive assessment through electrocardiogram and respiratory recordings.^29^^,^^30^^,^^31^ However, research exploring its role in understanding the cardiorespiratory mechanisms underlying music perception across infancy remains limited.

One study examined how tonal and atonal music influenced RSA in 40 mothers and their 3-month-old infants, with the tonal fragment based on harmonic structures similar to those in mother-infant vocal interactions, while the atonal fragment lacked tonal organization.^32^ The tonal music fragment was based on harmonic structures resembling those found in mother-infant vocal interactions, whereas the atonal fragment lacked tonal organization. The findings revealed that infants exhibited significantly higher RSA levels when exposed to tonal music compared to both the atonal fragment and baseline conditions, which could be interpreted as enhanced vagal activity in response to tonal stimuli.^32^ The findings showed higher infant RSA levels with tonal music, possibly reflecting enhanced vagal activity. Moreover, RSA responses differed between infants and mothers, with less synchronization when their cardiovascular systems were not mutually influencing each other, highlighting the role of physiological co-regulation in musical contexts.

In adults, research controlling for the respiratory component of RSA suggests that music’s effect on autonomic function is strongly influenced by its valence and arousing properties.^33^ Most of the studies investigating physiological responses to music in adults are based on heart rate (HR) or heart rate vaiability (HRV) measures, but findings remain inconsistent, reflecting the complexity of autonomic regulation in reaction to emotional auditory stimuli.^34^ Arousing^35^ or calming effects^36^ have been found, but the relationship between HR changes and musical characteristics is not always straightforward. Listening to stimulating music, as opposed to calming music, seems to be linked to a decrease in RR series variability, reflected in the reduction of both low-frequency and HF power, alongside an increase in HR.^37^ Additionally, when music elicits strong emotional responses, heightened physiological arousal has been found.^38^

Beyond RSA, other mechanisms such as cardiorespiratory phase coupling and synchronization capture additional aspects of the coordination between heart rate and breathing.^39^^,^^40^ Phase coupling describes coordinated phase relationships between heart rate and respiration, independent of RSA, and is particularly relevant in preterm infants with developing cardiorespiratory regulation.^41^^,^^42^ Its maturation over time offers critical insights into autonomic and cardiorespiratory development,^43^ motivating its inclusion in the present study for a more comprehensive view. Additionally, we aimed to address directionality aspects in cardiorespiratory interactions. While previous methods have described the overall strength of heart-respiration coupling, they do not capture directionality—that is, influences flow primarily from respiration to the heart (classical RSA) or from the heart to respiration (non-respiratory RRi modulations). Investigating directionality in preterm newborns may help clarify developmental differences compared to adults.^41^^,^^44^

This study aims to investigate how music influences cardiorespiratory dynamics in preterm infants and adults during rest and music listening. A sound environment for preterm infants was specially composed by Andreas Vollenweider, incorporating instruments such as the Indian flute (punji), harp, and bells, over a continuous harmonic background.^6^ We analyzed heart rate variability and respiration time series to examine the mechanisms and directionality of music-induced autonomic changes in both groups. We hypothesized that music would alter HF power of RRi series, reflecting autonomic shifts, and that preterm infants will show distinct patterns of coupling and directionality compared to adults, consistent with different stages of autonomic maturation.

## Results

### Music modulates preterm infant’s cardiac activity

Heart rate analysis, the gold standard for autonomic assessment, has been applied to quantify autonomic activation during music stimulation. Specifically, we extracted frequency-domain indices to quantify the autonomic nervous system (ANS) activity: the HF power as an index of purely parasympathetic activation and the low-to-high frequency power ratio (LF/HF) as an index of physiological arousal.^45^

**Figure 1:**
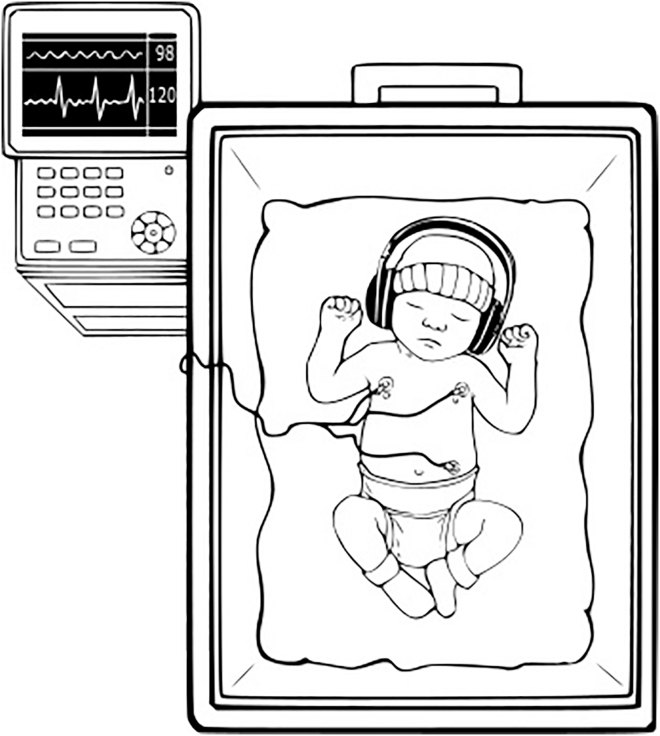
Representation of the acquisition setup The infant was placed in the NICU incubator, wearing size-adjusted headphones, while physiological signals were acquired using electrodes in a 3-lead configuration.

**Figure 2:**
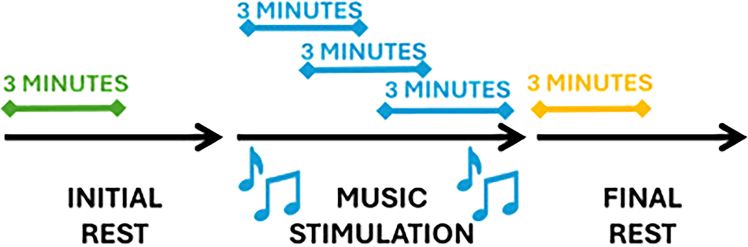
Temporal representation of the protocol steps

**Figure 3:**
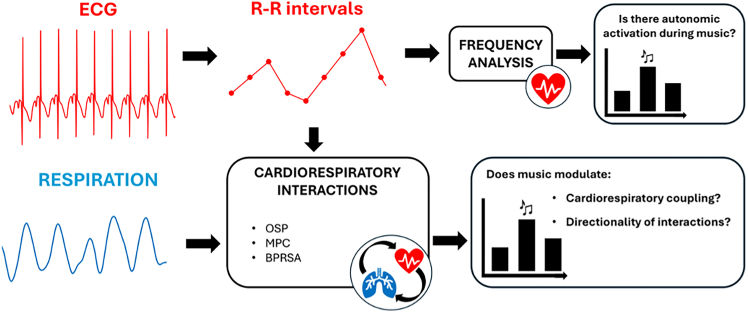
Schema of the performed analysis

**Figure 4:**
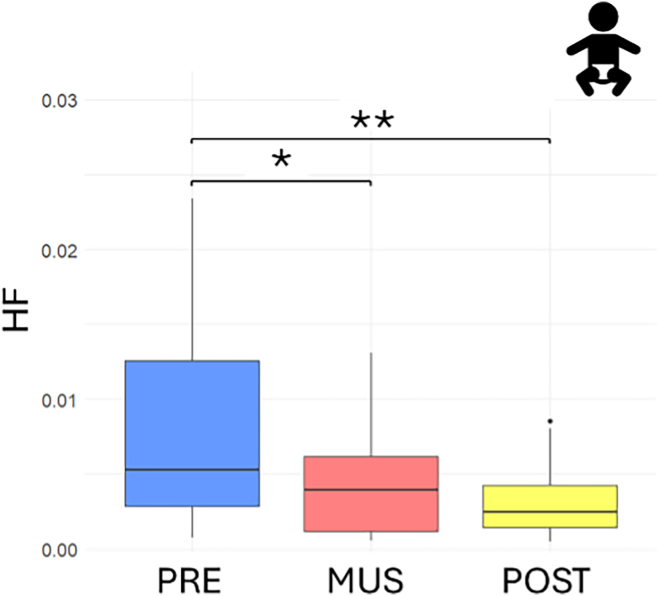
Boxplot representing the HF power for preterm infants (*n* = 18) before (blue), during (red), and after (yellow) music stimulation Significance thresholds are represented by ∗*p* < 0.05, ∗∗*p* < 0.01.

A representation of the set-up is presented in Figure 1. The timeline of the experimental protocol is shown in Figure 2, whereas Figure 3 presents on an overall scheme of the analyses performed. In preterm infants, a significant change in HF was observed throughout the experimental protocol (Friedman’s test, χ^2^(2) = 10.111, *p* = 0.006), with a notable decrease from the initial baseline to the music condition (V = 146, Bonferroni-corrected *p* value = 0.020) and to the post condition (V = 152, Bonferroni-corrected *p* value = 0.007). Figure 4 shows the boxplots of the HF power related to the three experimental conditions. No significant change was found for the LF/HF ratio (Friedman’s test, χ^2^(2) = 1.7778, *p* value = 0.411). Results in the form of mean and standard deviations have been reported in Table 1. Population characteristics of the preterm infants have been reported in Table 2.

**Table 1:** Summary of metrics values obtained for infants during, before, and after the music protocol

Metric	Description	Pre		Music		Post	
		Mean	Std	Mean	Std	Mean	Std
**Infants**							

HF	Power in the high-frequency band (0.2–1.5)	0.008204	0.007212	0.004539	0.003851	0.00321	0.002383
LF/HF	Ratio of low-to-high frequency power	9.779873	5.448994	11.04703	5.370513	9.37231	7.833609

**Table 2:** Characteristics of the infant population

Characteristics	Value
Population, *N*	18
GA at birth (weeks), (mean ± std)	27.63 ± 2.24
Birthweight (g), (mean ± std)	974.56 ± 379.66
GA at test (week), (mean ± std)	37.73 ± 0.80

Note: GA, gestational age.

### Music modulates adult’s cardiac activity

**Figure 5:**
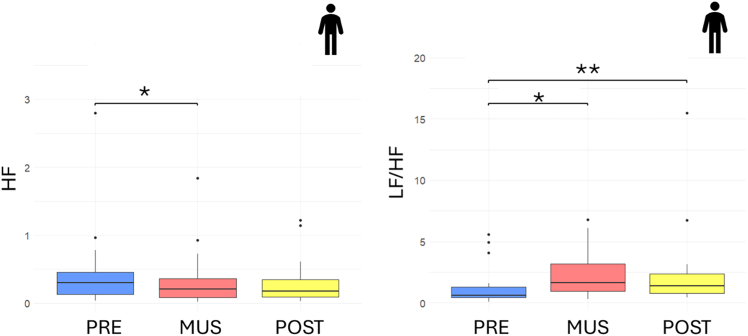
Boxplot representing the HF power (left) and the HF/LF ratio (right) for adults (*n* = 19) before (blue), during (red), and after (yellow) musical stimulation Significance thresholds are represented by ∗*p* < 0.05, ∗∗*p* < 0.01.

In the group of adults, a statistically significant change in HF power was observed (Friedman’s test, χ^2^(2) = 9.7895, *p* value = 0.007), with a marked decrease from the initial baseline to the music condition (V = 160, Bonferroni-corrected *p* value = 0.021). Additionally, the LF/HF ratio values exhibited significant changes across the three phases (χ^2^(2) = 15.579, Friedman’s test, *p* value = 4.141e−4), showing a significant increase from the initial baseline to the music condition (V = 25, Bonferroni-corrected *p* value = 0.010) and from the initial baseline to the post condition (V = 23, Bonferroni-corrected *p* value = 0.007). The results are displayed in Figure 5, while all mean and standard deviations have been reported in Table 3. Population characteristics of the adults have been reported in Table 4.

**Table 3:** Summary of metrics values obtained for adults during, before, and after the music protocol

Metric	Description	Pre		Music		Post	
		Mean	Std	Mean	Std	Mean	Std
**Adults**							

HF	Power in the high-frequency band (0.15–0.4)	0.448345	0.621802	0.331333	0.438565	0.304203	0.346088
LF/HF	Ratio of low-to-high frequency power	1.323374	1.646637	2.312951	1.883765	2.455771	3.468948

**Table 4:** Characteristics of the adult population

Characteristics	Value
Population, *N*	19
Sex, M |F	7 | 12
Age range 18–20 years, *N*	5
Age range 21–30 years, *N*	14
Music practice, yes | no	12 | 7

### Music increases RSA and cardiorespiratory coupling in hospitalized preterm infants

In the infant population, P_x_ demonstrated a significant increasing trend across the different phases (Friedman’s test, χ^2^(2) = 10.778, *p* value = 0.005). In particular, the strength of cardiorespiratory coupling increased significantly from the initial baseline to the post condition (V = 18, Bonferroni-corrected *p* value = 0.006). The mean phase coherence (MPC) displayed a similar increasing trend (Friedman’s test, χ^2^(2) = 6.3333, *p* value = 0.042); however, multiple comparisons were not significant after Bonferroni’s correction between the initial and the post condition (V = 37, Bonferroni-corrected *p* value = 0.103).

**Figure 6:**
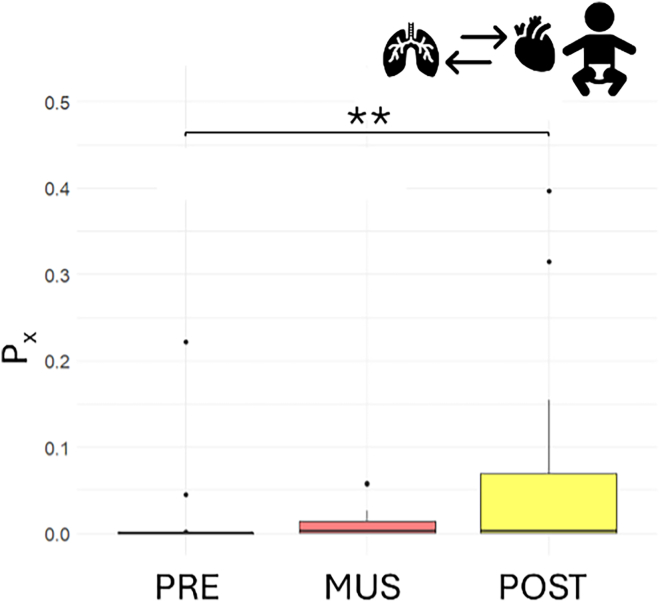
Boxplot representing the P_x_, for infants before (blue), during (red), and after (yellow) musical stimulation Significance thresholds are represented by ∗*p* < 0.05, ∗∗*p* < 0.01.

The boxplots of P_x_ distributions in the three experimental sessions in preterm infants are displayed in Figure 6. All mean and standard deviations have been reported in Table 5.

**Table 5:** Summary of cardiorespiratory coupling assessment for infants during, before, and after the music protocol

Metric	Description	Pre		Music		Post	
		Mean	Std	Mean	Std	Mean	Std
**Infants**							

P_x_	the power related to the respiration component with respect to the total power of the RRi signal	0.015174	0.052612	0.011649	0.01833	0.062765	0.115133
MPC	mean phase coherence between RRi and respiration	0.051738	0.039092	0.07347	0.066613	0.095348	0.085693
MI – RRi (acceleration) → Resp	mutual information between trigger (RRi acceleration) and target (respiration)	0.073218	0.034821	0.109471	0.071592	0.127381	0.062722
MI – RRi (deceleration) → Resp	mutual information between trigger (RRi deceleration) and target (respiration)	0.079148	0.032712	0.113008	0.060013	0.102515	0.058334
xcorr – RRi (acceleration) → Resp	cross-correlation between trigger (RRi acceleration) and target (respiration)	0.206282	0.081108	0.21941	0.081268	0.293482	0.182323
xcorr – RRi (deceleration) → Resp	cross-correlation between trigger (RRi deceleration) and target (respiration)	0.198205	0.066926	0.197108	0.068263	0.290639	0.14881
MI – Resp (max) →RRi	mutual information between trigger (respiration max) and target (RRi)	0.087447	0.039752	0.139976	0.106918	0.120287	0.099248
MI – Resp (min) → RRi	mutual information between trigger (respiration min) and target (RRi)	0.089159	0.028408	0.13978	0.115719	0.125236	0.100091
xcorr – Resp (max) → RRi	cross-correlation between trigger (respiration max) and target (RRi)	0.121213	0.078226	0.186635	0.125617	0.205467	0.185825
xcorr – Resp (min) → RRi	cross-correlation between trigger (respiration min) and target (RRi)	0.134169	0.107247	0.197699	0.13704	0.195232	0.181676
MSCHOERE	mean of magnitude-squared coherence estimate	0.519196	0.032492	0.520493	0.042576	0.516125	0.046694

The bivariate phase-rectified signal averaging (BPRSA) technique quantifies quasi-periodic oscillations in a time series due to interactions with another series. Coupling between signals has been measured using a linear metric, the maximum value of the cross-correlation function (max_xcorr), and a nonlinear marker, the mutual information (MI).

**Figure 7:**
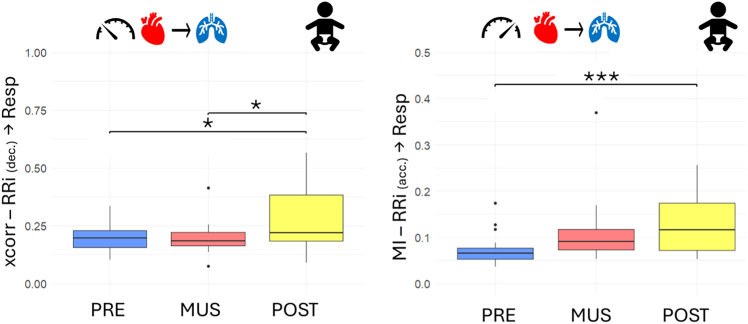
Boxplot depicting the BPRSA results for infants in NICU before (blue), during (red), and after (yellow) musical stimulation On the right, the relationship between the heart acceleration and the respiration; on the left, the relationship between the heart deceleration and the respiration. Significance thresholds are represented by ∗*p* < 0.05, ∗∗*p* < 0.01,∗∗∗*p* < 0.001.

The BPRSA was used to firstly quantify the strength of the influence of RR series changes on respiration using heart rate acceleration as anchor points. Relevant trends are represented in Figure 7. The MI values displayed an increasing trend during the experiment (Friedman’s test, χ^2^(2) = 11.444, *p* value = 0.003), with the multiple comparison tests being statistically significant between the initial and the post condition (V = 10, Bonferroni-corrected *p* value = 0.980e−3). The max_xcorr metrics computed between the two series reported a matching increasing trend; however, the difference is not significant (Friedman’s test, χ^2^(2) = 5.3333, *p* value = 0.069).

Concerning the effects investigated during the deceleration of the heart rate, a significant difference (Friedman’s test, χ^2^(2) = 7, *p* value = 0.030) was found in the post condition, which displayed values significantly higher than both the initial baseline (V = 30, Bonferroni-corrected *p* value = 0.042) and the music condition (V = 27, Bonferroni-corrected *p* value = 0.027). No significant differences are reported for the MI values after the multiple comparisons’ correction (Friedman’s test, χ^2^(2) = 6.3333, *p* value = 0.042).

BPRSA coupling in the direction from respiration to the heart during minimum and maximum impedance presented no statistically significant change during our experimental paradigm.

### Music decreases RSA and cardiorespiratory coupling in adults

**Figure 8:**
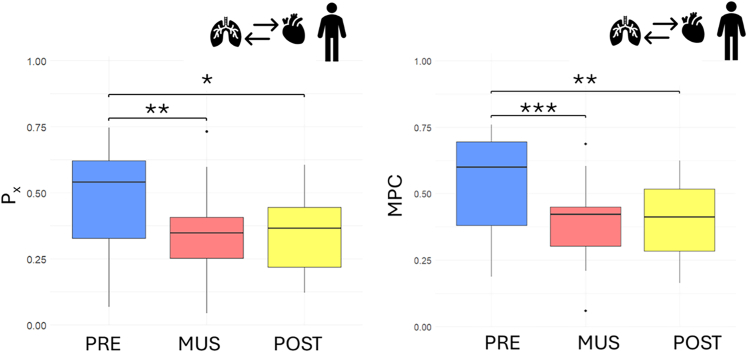
Boxplot depicting the P_x_ (left) and MPC (right) results for adults before (blue), during (red), and after (yellow) musical stimulation Significance thresholds are represented by ∗*p* < 0.05, ∗∗*p* < 0.01,∗∗∗*p* < 0.001.

In the adult population, P_x_ showed a significant decrease (Friedman’s test χ^2^(2) = 11.789, *p* value = 0.003) from initial baseline to the music condition (V = 173, Bonferroni’s corrected *p* value pre vs. music = 0.002) and between initial baseline and post condition (V = 164, Bonferroni’s corrected *p* value pre vs. music = 0.012). No significant differences were reported between the music condition and the post condition. The related boxplots are displayed in Figure 8. The MPC results are consistent, showing the same marked decrease (Friedman’s test, χ^2^(2) =, 15.895 *p* value = 0.353 e−3), from the initial resting state session to the music condition (V = 179, Bonferroni’s corrected *p* value pre vs. music = 0.630e−3) and between the initial baseline and post condition (V = 175, Bonferroni’s corrected *p* value = 0.157 e−2).

Regarding BPRSA, no significant change has been detected for any parameters (Friedman’s test, max in cross-correlation RRi to respiratory series (RESP) during heart rate acceleration χ^2^(2) = 3.2632, *p* value = 0.195; during heart rate deceleration χ^2^(2) = 4.5263, *p* value = 0.104; MI during heart rate acceleration χ^2^(2) = 2.2105, *p* value = 0.331; during heart rate deceleration χ^2^(2) = 0.42105, *p* value = 0.810). The trends of max_xcorr are shown to be consistent with previously reported results. All mean and standard deviations have been reported in Table 6.

**Table 6:** Summary of cardiorespiratory coupling assessment for adults during, before, and after the music protocol

Metric	Description	Pre		Music		Post	
		Mean	Std	Mean	Std	Mean	Std
**Adults**							

P_x_	the power related to the respiration component with respect to the total power of the RRi signal	0.480474	0.187891	0.338614	0.161297	0.351352	0.139318
MPC	mean phase coherence between RRi and respiration	0.554645	0.174877	0.399883	0.148792	0.392966	0.136645
MI – RRi (acceleration) → Resp	mutual information between trigger (RRi acceleration) and target (respiration)	0.336608	0.367421	0.232035	0.189881	0.253661	0.22115
MI – RRi (deceleration) → Resp	mutual information between trigger (RRi deceleration) and target (respiration)	0.326791	0.320487	0.247504	0.22466	0.255173	0.251278
xcorr – RRi (acceleration) → Resp	cross-correlation between trigger (RRi acceleration) and target (respiration)	0.788823	0.203796	0.695743	0.17845	0.706892	0.209103
xcorr – RRi (deceleration) vResp	cross-correlation between trigger (RRi deceleration) and target (respiration)	0.799131	0.193346	0.710285	0.181315	0.682189	0.218088
MI – Resp (max) → RRi	mutual information between trigger (respiration max) and target (RRi)	0.316509	0.32179	0.317323	0.259399	0.255873	0.256091
MI – Resp (min) → RRi	mutual information between trigger (respiration min) and target (RRi)	0.364372	0.359241	0.319997	0.268196	0.276864	0.323218
xcorr – Resp (max) → RRi	cross-correlation between trigger (respiration max) and target (RRi)	0.845897	0.13509	0.832187	0.14833	0.809727	0.166206
xcorr – Resp (min) → RRi	cross-correlation between trigger (respiration min) and target (RRi)	0.873634	0.083933	0.828669	0.159192	0.795186	0.181538
MSCHOERE	mean of magnitude-squared coherence estimate	0.423489	0.072932	0.393902	0.056347	0.365997	0.057784

### Comparison with standard coherence measurement

To facilitate comparison with a more standardized method, we additionally computed the magnitude-squared coherence (MSCHOERE) between the respiratory and RRi signals, as a linear measure of coupling. The resulting values are reported as follows.

**Figure 9:**
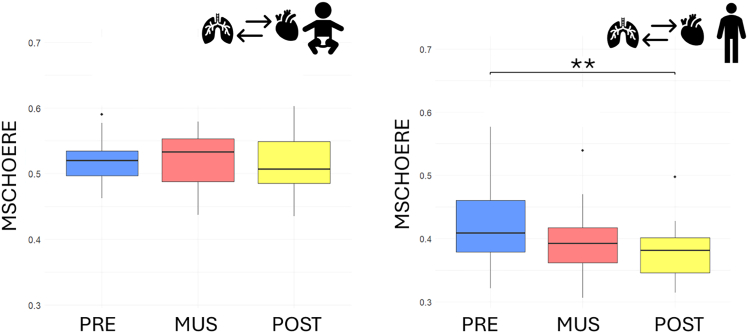
Boxplot depicting the MSCHOERE value for infants (left) and adults (right), before (blue), during (red), and after (yellow) musical stimulation Significance thresholds are represented by ∗*p* < 0.05, ∗∗*p* < 0.01.

In the group of adults (Figure 9, right), a statistically significant decrease in magnitude square coherence was observed (Friedman’s test, χ^2^(2) = 6.421, *p* value = 0.040), with a marked decrease from the initial baseline to the post condition (V = 166, Bonferroni-corrected *p* value = 0.009).

In the group of preterm infants (Figure 9, left), no significant change in MSCHOERE was observed (Friedman’s test, χ^2^(2) = 0.4444, *p* value = 0.801).

### Main directionality of influence between cardiac and respiratory dynamics

To evaluate differences in the directionality of interactions, we used a novel approach based on the computation of cross-correlation and MI function on the BPRSA trigger and the target series (see STAR Methods). The BPRSA indices of coupling strength from RRi to respiration during heart rate acceleration and deceleration were compared with those from respiration to RRi during minimum and maximum impedance, for each phase.

**Figure 10:**
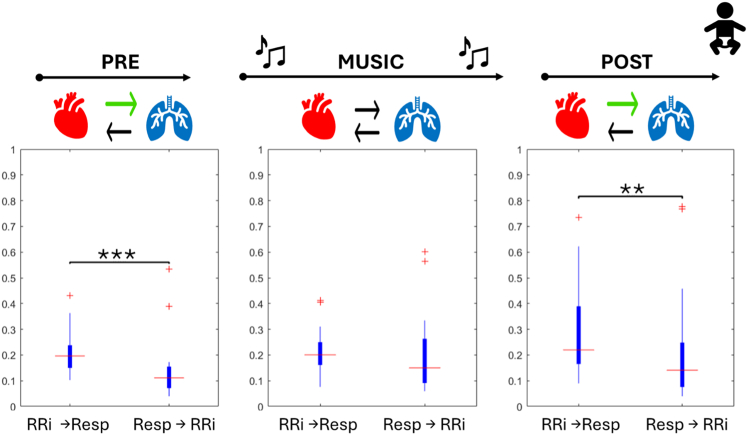
Directionality analysis for infants in NICU during the protocol Boxplots depict the aggregation of cross-correlation BPRSA values for each direction. Significance thresholds are represented by ∗*p* < 0.05, ∗∗*p* < 0.01,∗∗∗*p* < 0.001. In the top line, protocol phases are represented, and arrow depicting a significantly stronger directionality is highlighted in green.

For premature infants, the analysis compounded of cross-correlation values assessing the strength of interactions between RRi and respiration across conditions, comparing the two possible directions of interaction, revealed significant differences in coupling direction. A stronger coupling from RRi to respiration was observed during the initial baseline (V = 594, Bonferroni-corrected *p* value = 2.978 e^−5^) and the post condition (V = 532, Bonferroni-corrected *p* value = 0.004). However, during music stimulation, no significant differences were found (V = 378, Bonferroni-corrected *p* value = 1). Trends and relevant directions are depicted in Figure 10.

**Figure 11:**
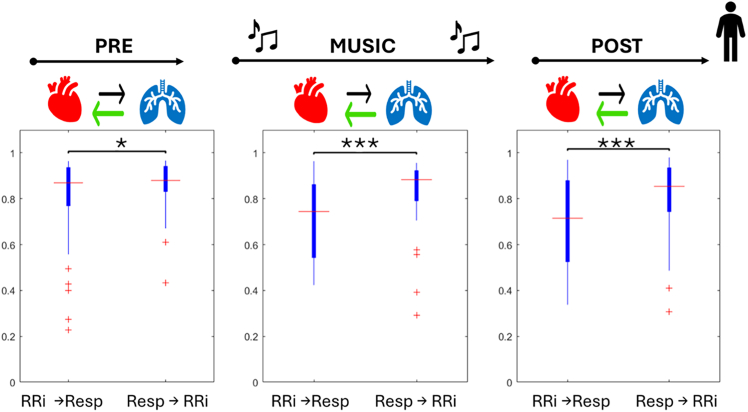
Directionality analysis for adults during the protocol Boxplots depict the aggregation of cross-correlation BPRSA values for each direction Significance thresholds are represented by ∗*p* < 0.05, ∗∗*p* < 0.01,∗∗∗*p* < 0.001. In the top line, protocol phases are represented, and arrow depicting a significantly stronger directionality is highlighted in green.

In adults, the analysis of cross-correlation values revealed significant differences in the strength of coupling directions (RRi to respiration vs. respiration to RRi) across all phases of the protocol, with stronger coupling from respiratory activity to heart activity. This difference was observed during the initial baseline (V = 189, Bonferroni-corrected *p* value = 0.023), the music phase (V = 84, Bonferroni-corrected *p* value = 2.376 e^−5^), and the post condition (V = 131, Bonferroni-corrected *p* value = 0.897 e^−3^). Trends and relevant directions are depicted in Figure 11.

For both preterm infants and adults, the MI-based approach did not highlight statistically significant differences between the various phases of the experimental protocol, except for the music elicitation phase in adults where a consistent respiration to RRi flow was found.

## Discussion

The goal of our study was to explore how the cardiac and respiratory systems adjust their rhythms and dynamics to a music stimulus in preterm newborns compared to adults. In particular, we aimed to examine different aspects of cardiovascular coupling to disentangle the underlying physiological processes that change during development.

In preterm infants, the observed decrease in HF power accompanied by an increase in RSA suggests a shift toward an attentive and emotionally engaged state during music listening, potentially reflecting a reorganization of autonomic function to support better self-regulation. This pattern is consistent with the notion of calm alertness, where increased RSA prepares infants for interaction with their environment, including auditory stimuli.^46^

Our findings align with previous evidence that greater vagal tone promotes more efficient homeostatic regulation^47^ and supports favorable developmental outcomes such as improved attachment, reciprocity, executive function, and adaptive autonomic profiles.^48^ From a psychophysiological perspective, higher RSA has been associated with better social engagement and flexible self-regulation,^49^^,^^50^^,^^51^ and our results suggest that music might facilitate this process even in a medically fragile population.

However, it is important to note that RSA is not a perfect marker of parasympathetic tone in isolation, as it can be influenced by respiratory patterns, metabolic demands, and developmental factors.^52^^,^^53^ These considerations underscore the need for cautious interpretation and the integration of respiratory parameters when drawing conclusions about vagal function from RSA data. Nevertheless, our results provide new insights into how music may help reorganize autonomic states in preterm infants, potentially supporting early social and cognitive development.

The differing autonomic responses to music observed between adults and preterm infants in this study may reflect differences in motor engagement and the developmental status of the autonomic system. Our adult participants showed reduced HF power and RSA, consistent with previous evidence that music can elicit rhythmic movement or motor simulation, thereby shifting autonomic tone toward sympathetic dominance and promoting an active, aroused state.^54^

In contrast, preterm infants, who show a consistent motor response to rhythm around 7 months of age^55^ and who process instrumental sounds in regions beyond the sensorimotor system,^56^ did not exhibit comparable autonomic changes. These results extend previous findings by demonstrating that the physiological impact of music may depend on both motor readiness and autonomic maturity, highlighting that preterm infants may engage with music in ways that do not involve motor preparation but could nonetheless support other domains of neurodevelopment.

Thirdly, our results demonstrate a predominant vagal cardiac control in the cardiorespiratory dynamics of preterm infants, contrasting with a respiration-driven mechanism in adults. This supports existing theories of bidirectional heart-lung communication involving complex feedback loops modulated by mechanical factors, brainstem control, ANS activity, and baroreceptor and chemoreceptor feedback.^57^^,^^58^^,^^59^

Prior studies using both linear and nonlinear coupling methods^40^^,^^60^^,^^61^^,^^62^^,^^63^^,^^64^^,^^65^ consistently report respiration as the dominant driver of heart rhythms in adults, which aligns with our adult data. In contrast, our preterm cohort showed different regulatory patterns, likely reflecting ongoing ANS maturation and incomplete development of feedback mechanisms.^42^^,^^66^^,^^67^

While RSA and related couplings increase with developmental age,^44^ preterm infants often present variable or even absent RSA.^18^^,^^41^^,^^68^^,^^69^^,^^70^^,^^71^ The variability in RSA presence and strength reported in the literature^18^^,^^41^^,^^71^ may stem from its transient nature, suggesting that RSA is an evolving mechanism rather than a fully established one at preterm birth. The present findings extend previous work by showing that music-related modulation of directionality can help probe the fragile, evolving cardiorespiratory regulation in preterm infants, which is particularly susceptible to the NICU environment and its impact on parasympathetic development.^72^^,^^73^^,^^74^^,^^75^

Regarding phase synchrony assessed via MPC, our study showed that adults had decreased coupling values, consistent with their reduced RSA and reduced MSCHOERE between respiration and heart rate variability, while infants displayed a nonsignificant trend toward increased MPC and variable coherence patterns. This variability in infants likely reflects their still-maturing cardiorespiratory regulation, where phase synchronization is emerging and more difficult to measure robustly.^42^^,^^76^

These results extend earlier observations of bidirectional interactions in preterm infants,^68^^,^^77^ reinforcing that cardiorespiratory coordination in neonates differs from the respiration-driven interactions observed in adults. Our findings further reinforce this hypothesis, demonstrating that very preterm infants do not yet exhibit the stable respiratory-led interaction pattern characteristic of mature individuals. In line with Joshi et al.,^41^ our data highlight a stronger heart-to-respiration influence in preterm infants, in contrast to the mature respiration-to-heart pattern consistently observed in adults.

Finally, our observation of increased RSA after music exposure, together with a shift toward more balanced coupling, suggests that music might support the development of more stable autonomic interactions in this vulnerable group, echoing proposals from intervention studies.^68^^,^^78^

### Limitations of the study

To assess the progressive maturation of the cardiorespiratory system through this multi-level analysis of responses to musical stimuli, it is essential to cover critical ages across development. First, two distinct age ranges in preterm development—before 32 weeks of gestational age and term age—will be particularly interesting to be investigated. The progressive maturation of the cardiorespiratory system during a preterm infant’s stay in the NICU is influenced by both intrinsic maturation mechanisms and environmental factors. Determining whether a music intervention could progressively support this maturation is crucial for developing early intervention guidelines in this at-risk population.

A further consideration concerns the lack of direct comparative data from healthy term-born infants under identical experimental conditions. Burtchen et al. (2019)^79^ reported that late-preterm infants (35–36 weeks gestation) continue to exhibit distinct autonomic patterns at term-equivalent age compared to full-term newborns, with persistently higher heart rates and less mature heart rate variability. This suggests that prematurity leaves a lasting effect on autonomic function, even after the infant reaches term-equivalent age.

Similarly, Souza Filho et al. (2019)^80^ observed that preterm infants tend to show lower heart rate complexity and reduced parasympathetic activity relative to term-born peers, consistent with delayed autonomic maturation. Although these heart rate measures gradually improve, they may not fully align with those of term-born infants of the same postmenstrual age. Taken together, the present results we observed during music stimulation may reflect a unique autonomic trajectory linked to early extrauterine experience and atypical sensory environments associated with prematurity. In contrast, healthy term-born infants typically follow a more synchronized autonomic and sensory development within the intrauterine environment and may not demonstrate the same response pattern to the musical stimuli.

Additionally, this paper primarily focused on cardiorespiratory mechanisms in preterm infants, with adults included mainly as a comparative model to illustrate mature physiological responses. However, we acknowledge the importance of considering prior musical training and aptitude when interpreting the adult data, as these factors may influence the strength of the observed responses. Although the current sample is too small to assess the impact of musical background, this will be explored in future studies.

Our findings offer new insights into the development of cardiorespiratory interactions in preterm infants, highlighting the similarities and differences of their autonomic regulation compared to adults. While cardiac frequency domain responses to music were similar across both groups, a more detailed analysis of RSA and phase coupling revealed fundamental differences in the underlying mechanisms. These results carry interesting implications for neonatal care, particularly in the integration of music-based interventions within the NICU environment. By deepening our understanding of how music influences autonomic regulation in preterm infants, this study reinforces the potential benefits of auditory enrichment in neonatal care. Future work should also assess the practical implementation of tailored soundscapes in the NICU to promote optimal autonomic and neurophysiological development, potentially reducing the risks associated with autonomic dysfunction in this vulnerable population.

## Resource availability

### Lead contact

Further information and requests for resources and reagents should be directed to and will be fulfilled by the lead contact, Manuela Filippa (manuela.filippa@unige.ch).

### Materials availability

This study did not generate new unique reagents.

### Data and code availability

• Data are available upon request.
• This article does not report original code.
• Any additional information required to reanalyze the data reported in this article is available from the lead contact upon request.

## Acknowledgments

This work was supported by the 10.13039/501100001711Swiss National Science Foundation (FNS) (Principal Investigator: Prof. H.P., grant ID: F02-12516), as well as the Dora Foundation, Prim’Enfance Foundation, K Foundation, and the Von Meissner Foundation.

## Author contributions

F.M., H.P., B.M.F., and B.D. contributed to conceptualization and experimental design. B.D., L.L., and B.M.F. carried out data collection. L.L., S.E.P., and N.M. performed physiological data analysis and contributed to interpretation. V.A. and H.S. provided the music and voice extracts. B.M.F., H.P. provided clinical oversight and participant access. F.M., H.P., and N.M. supervised the project. L.L. and F.M. drafted the manuscript with input from all co-authors. All authors read and approved the final version of the manuscript.

## Declaration of interests

The authors declare no competing interests.

## STAR★Methods

### Key resources table

**Table:** 

REAGENT or RESOURCE	SOURCE	IDENTIFIER
**Biological samples**		

21 infants	Neonatal Intensive Care Unit (NICU) at the University Hospitals of Geneva, Switzerland	
20 adults	University of Geneva	

**Software and algorithms**		

MATLAB (R2021b)	MathWorks Inc, Natick, MA, USA	https://it.mathworks.com/products/matlab
Kubios HRV Standard (v2.2)	Zhang et al.^81^	https://www.kubios.com/
R (version 4.2.2)		https://www.r-project.org/

### Experimental model and study participant details

#### Ethics statement

The Swiss Research Ethics Committee approved the study (BASEC no. 2023-00718), and written informed consent was obtained from all parents.

#### Human participants

A total of 21 infants born before 32 weeks of gestation were recruited from the Neonatal Intensive Care Unit (NICU) at the University Hospitals of Geneva, Switzerland. Preterm infants with major brain lesions, such as grade III intraventricular hemorrhage with or without periventricular hemorrhagic infarction, or cystic periventricular leukomalacia, were excluded. In addition, no genetic syndromes were present in the analyzed cohort. In terms of clinical course, approximately one-third of the preterm infants born before 32 weeks of gestational age are diagnosed with bronchopulmonary dysplasia. It is important to note, however, that in the present cohort, this was classified as mild, with no need for ongoing respiratory support at the time of the data collection. None of the infants received caffeine treatment at the moment of the assessment. No further severe neonatal complications were reported as exclusion criteria beyond those described.

During the data acquisition, one infant was excluded due to excessive fussiness, and two were excluded due to instrumentation issues.

Infant’s demographic data for the final population are presented in Table 2.

Additionally, a cohort of 20 adults was recruited to undergo the same music protocol as the infants. One adult was excluded due to instrumentation issues.

Adult’s demographic data are reported in Table 4.

### Method details

#### Experimental procedure

While in intensive care, infants were exposed to music intervention via headphones Sennheiser PXC 250-II (Sennheiser electronic GmbH & Co.KG, Wedemark, Germany), adapted to the size of the baby's head. During the whole protocol physiological signals were acquired. A representation of the set-up can be seen in Figure 1.

A baseline period of at least 3 minutes was acquired before and after the music stimulation, defined as a period in which infant was wearing the headphones without music was otherwise unstimulated. The music stimulation consisted in 6 minutes of stereophonic musical excerpts in B major, specifically created by the musician Andreas Vollenweider, with a sound level from 30dBA for the background to 65dBA for the peak with bells.

The music has been used in similar protocol in.^6^^,^^12^ The music piece is built on a harmonic foundation of human voices, occasionally enriched by harp, punji, and bells. The landscape is stereophonic, with instrument appearing intermittently. The main elements are the harp, introducing a soft texture and melodic emphasis, while the punji, a traditional Indian flute, carrying the principal melody and bells providing sporadic percussive accents in the harmonic structure.

##### Electrophysiological recording

Infant physiological signals were recorded using standard cardiorespiratory monitoring with the IntelliVue MP40 monitor (Philips Medical System, Eindhoven, Netherlands). The ECG signal was then automatically recorded using the IxTrend 2.1 software (Ixellence GmbH, Wildau, Germany; ECG was acquired at 500 Hz while the respiratory signal was acquired at 62.5 Hz. The adult’s ECG signals were recorded using the EGI’s Geodesic EEG System at a sampling frequency of 1000 Hz.

#### Data preprocessing

##### ECG pre-processing

First the time intervals between successive R-peaks were derived from the ECG signals using the Kubios 2.2 software,^82^ performing a visual inspection and artifact correction to ensure signal quality. Next, the resulting RR series were detrended with the smoothness priors’ approach,^83^ setting λ = 500, and resampled at 4Hz using a piecewise cubic spline interpolation. This produced a time series with even sampling and removed very low-frequency that could bias the following analysis. The resulting uniformly sampled series will henceforth be called RRi signals.

##### Infants’ respiratory signals preprocessing

Infants’ respiratory signals were pre-processed following.^81^ Signals were downsampled at 16 Hz filtered with a 5th-order Butterworth filter ([0.05–2] Hz), baseline-corrected using a 1.2s median trend removal, resampled to 4 Hz, and normalized via z-score.

##### Adults’ respiratory signals preprocessing

For adults, since breath signals were not available, the respiratory activity was derived from the ECG using the slope-range method.^84^ The ECG-derived respiratory signal (ECG-DR) served as a validated surrogate for the respiratory signal in adults.^17^ ECG signals were filtered with a twelve-order high-pass Butterworth filter (0.8 Hz) to remove baseline wander and a twelve-order low-pass Butterworth filter (30 Hz) to eliminate high-frequency noise. The slope range was defined as the difference between the maximum up-slope and the minimum down-slope within the QRS interval and used to represent respiratory activity, for details see.^84^ An outlier correction procedure was performed according to Bailon et al. (2006),^85^ and signals were resampled at 4 Hz using cubic spline interpolation to match the RRi series.

#### Data analysis

##### Window selection

Autonomic activity and cardiorespiratory coupling were analyzed in three stages: before, during, and after musical elicitation. Three-minute windows were used: initial baseline, three overlapping windows during stimulation, and post condition (see Figure 2). For music elicitation, the average of the feature values over the three overlapping windows was computed to characterize the global effect.

##### RRi frequency analysis

The autonomic modulation on RRi was investigated by using the standard frequency analysis and adding the information obtained from the application of the Orthogonal Subspace Decomposition (OSP).^17^^,^^86^

##### Standard frequency analysis

The power density spectrum (PDS) of RRi signals was computed for each three-minute window using Welch's method. RRi signal power was computed in two frequency bands: LF and HF, with age-specific ranges—[0.04–0.15] Hz (LF) and [0.15–0.4] Hz (HF) for adults, and [0.02–0.2] Hz (LF), and [0.2–1.5] Hz (HF) for infants, according to.^87^^,^^88^ HF power along with the LF/HF ratio were calculated. HF power reflects parasympathetic modulation.^45^^,^^89^ LF/HF ratio has been interpreted as potential index of physiological arousal, since its original interpretation of it as a marker of sympatho -vagal balance has been debated,^90^^,^^91^ citing the complex, non-linear, and often non-reciprocal interactions between the sympathetic and parasympathetic systems.

##### Orthogonal subspace decomposition (OSP)

To identify factors influencing RRi changes, the OSP method^17^^,^^86^ was applied. OSP separates respiratory components linearly related to RRi from residual ones, such as sympathetic and vagal modulations unrelated or non-linearly related to respiration. This technique produces two signals: one influenced by the respiratory activity and another for residual activity.

The RRi signal is projected into two orthogonal subspaces, representing the respiratory activity and the residual activity respectively, as follows. Given a respiratory signal X=x(1),…,x(N), and the RRi signal Y=y(1),…,y(N), and the model order m, a subspace V defined by all variations in X is constructed using X and its delayed version from 1 to m samples:

$$V=\left[X_{0},X_{1},\ldots ,X_{m}\right]$$

The vectors X_d_ are defined as:Xd=[x(d),x(d+1),x(d+2),…,x(N−m+d)],T where (d=0,…,m)

Using this subspace, a projection matrix P is defined as:

$$P=V{\left(V^{T}V\right)}^{-1}V^{T}$$

Then Y is projected over the respiratory subspace using P, and Y_X_, the RRi component linearly related to respiration is obtained as:

$$Y_{X}=PY$$

The residual is calculated as the orthogonal component:

$$Y_{⊥}=Y-Y_{X}$$

The model order m has been defined as the minimum number of delays obtained considering the minimum description length (MDL) principle and the Akaike Information Criterion (AIC).

##### Cardiorespiratory analysis

Combining the information extracted from the RRi and the respiratory signals, we applied three approaches to investigate the cardiorespiratory interaction: the relative power of the respiratory component obtained through the OSP,^17^^,^^86^ the Mean Phase Coherence (MPC),^92^ and the Bivariate Phase-Rectified Signal Averaging (BPRSA).^93^

##### OSP-derived relative power of the respiratory component

The percentage of the power related to the respiration component with respect to the total power of the RRi signal (hereinafter P_x_) was computed as an index of strength of cardiorespiratory coupling, quantifying the amount of information shared between respiration and heart rate variability dynamics.^17^^,^^28^

The metric P_x_ is calculated as:

$$P_{X}=\frac{Y_{X}^{\prime }Y_{X}}{Y^{\prime }Y}$$

This approach has been reported as one of the most effective to quantify RSA strength^28^ and used to assess the phenomena in clinical evaluations.^94^

##### Mean phase coherence (MPC)

The Mean Phase Coherence (MPC) quantifies the phase synchronization of two time-series regardless of their amplitude.

This synchronisation has been observed in chaotic systems and coupled complex oscillators.^95^^,^^96^ According to the previous literature, the MPC is suitable for application to time series expressing coupled physiological dynamics, such the heart and respiration activities, as they display clear nonlinear, complex behaviour^97^ and can act as weakly coupled complex systems.^98^

The MPC metric has been computed as described in.^92^ Given two time-series x and y:

$$\left|\varphi_{x}\left(t\right)-\varphi_{y}\left(t\right)\right|=\text{const}$$

with $\varphi_{x}\left(t\right)$ and $\varphi_{y}\left(t\right)$ representing the instantaneous phases of x and y respectively, the final metrics is computed as follows

$$MPC=\left|\frac{1}{N}\sum_{j=0}^{N-1}e^{i\left(\varphi_{x}\left(j\Delta t\right)-\varphi_{y}\left(j\Delta t\right)\right)}\right|$$

where t is the time resolution, N is the sample number of each signal.

Phase-synchronization of cardiorespiratory series has been previously observed in the literature in adults,^98^ and in newborn infants.^76^

##### Bivariate Phase-Rectified Signal Averaging (BPRSA)

The Bivariate Phase-Rectified Signal Averaging approach^93^ quantifies the inter-relation between two signals, independently from non-stationarity and noise.

It detects anchor points $x_{i1},x_{i2},\ldots ,x_{iM}$ on a trigger signal X and their respective timing and averages corresponding fixed-length windows (2L) on target signal Y, yielding a modulated signal. The result is a signal of length 2L, representing the modulations on the target signal attributed to periodicities in the trigger. Given a total of M anchor points, the trend is defined as:

$${\text{BPRSA}}_{X\to Y}\left(k\right)=\bar{y}\left(k\right)=\frac{1}{M}\sum_{v=1}^{M}y_{i_{v}+k},k=-L,\ldots ,0,\ldots ,L-1$$

Respiratory and RRi time series (without detrending) were analyzed using L=55 samples in both interaction directions.

For the first direction For RRi as the trigger, anchor points corresponded to heart rate acceleration and deceleration, where deceleration was defined as:

$$\frac{1}{T}\sum_{j=0}^{T-1}x_{i+j}>\frac{1}{T}\sum_{j=1}^{T}x_{i-j}$$

given x the value of the RRi series, as such a deceleration in cardiac activity corresponds to an increase in the RR time intervals. For these computations T has been set to 1, as done in Joshi et al. (2019).^41^

For the reverse direction, the respiration time series has been used as a trigger signal, selecting two types of anchor points, i.e., the maximum and the minimum of each respiratory cycle.

Then, the strength of the coupling between the trigger and the target has been computed using a novel approach, according to,^99^^,^^100^ using both linear (maximum absolute value of normalized cross-correlation) and nonlinear (Mutual Information, MI) methods (Fraser & Swinney, 1986) via the Kernel density estimation method.^101^

##### Complementary magnitude square coherence analysis (MSCHOERE)

The magnitude-squared coherence MSC^102^ a frequency-dependent measure ranging from 0 to 1 that quantifies the linear correlation between two signals, *x* and *y*, at each frequency.

Given two time-series x and y, the Coherence is computed as:

$$C_{xy}\left(f\right)=\frac{{\left|P_{xy}\left(f\right)\right|}^{2}}{P_{xx}\left(f\right)\cdot P_{yy}\left(f\right)}$$

Where $P_{xx}\left(f\right)$ correspond to the power spectral density of serie x as a function of frequency *f*. $P_{xy}\left(f\right)$ correspond to the cross power spectral density of x and y. The power spectrums have been computed using Welch’s periodogram method, and then the average value across the frequency range [0-2] Hz has been chosen to quantify the overall coherence between the two series (RRi and Respiratory series).

### Quantification and statistical analysis

Statistical analysis was used to compare the markers of autonomic dynamics during the elicitation protocol phases. The normality of the data distribution was checked using the Lilliefors test. Due to the detection of non-Gaussian distributions, the Friedman test was used to compare changes across phases (initial rest, music elicitation, final rest). When statistically significant differences were found (Friedman’s p-value < 0.05), post-hoc pairwise Wilcoxon tests with Bonferroni correction were computed.

#### Directionality assessment

For the BPRSA analysis, differences in coupling directionality were evaluated by comparing the coupling strength between RRi → respiration during acceleration/deceleration and respiration → RRi during minimum/maximum impedance for each phase. In the absence of a specific hypothesis for selecting the triggers, acceleration/deceleration for RRi → respiration and maximum/minimum for respiration → RRi were aggregated. This comparison aimed to explore directional dynamics (not causality) using both cross-correlation and Mutual Information (MI). Wilcoxon signed rank test for paired samples was used, with Bonferroni’s correction of p-values applied for multiple comparisons.

Data analyses were performed separately on the population of infants (number of participants = 18; characteristics summarized in Table 2) and on the population of adults (number of participants = 19; characteristics summarized in Table 4).

MATLAB software (see STAR Methods) was used for feature extraction and Gaussianity testing, while R software was used to perform statistical analyses (see STAR Methods).

A summary of the trends and significant comparisons is presented in the boxplots (Figures 4, 5, 6, 7, 8, 9, 10, and 11), while detailed results in the form of means and standard deviations for each protocol phase in each population are reported in Tables 1, 3, 5, and 6.
