# Supplementary material for: Cardiorespiratory mechanisms triggered during music perception in preterm infants and adults
Source: iScience. 2025 Sep 11;28(10):113524. doi: 10.1016/j.isci.2025.113524 (PMC12513323; doi:10.1016/j.isci.2025.113524)
Supplement: Document S1. Figures S1 and S2 and Tables S1 and S2 [file mmc1.pdf]

iScience, Volume *28*

Supplemental information

Cardiorespiratory mechanisms triggered

during music perception

in preterm infants and adults

Laura Lavezzo, Francisca Barcos-Munoz, Damien Benis, Juliette Margaria, Didier Grandjean, Enzo Pasquale Scilingo, Andreas Vollenweider, Stéphane Henriot, Mimma Nardelli, Petra Hüppi, and Manuela Filippa

SUPPLEMENTARY INFORMATION

To disentangle respiratory-related effects on the HRV dynamics from separate ANS control mechanisms, the Orthogonal Subspace Decomposition (OSP) approach was used (Varon et al., 2019), see details in Section(STAR Methods). For each frequency band (HF and LF), we extracted two components from HRV power spectrum: a respiratory component related to respiration (hereinafter RespComp), and a residual component (hereinafter Residual) associated to sympathetic and vagal modulations unrelated to breathing functions.

Table S1. Summary of metrics values obtained for infants before, during, and after the music protocol (Related to Table 1).

Infants

Metric	Description	pre		music		post	
		mean	std	mean	std	mean	std
HF RespComp	*Power in the high frequency band [0.2-1.5] for respiratory component*	0,00023	0,000613	0,000352	0,000725	0,00051	0,001112
LF/HF RespComp	*Ratio of low-to-high frequency power for respiratory component*	0,329849	1,273773	0,119846	0,216701	1,405746	3,744098
HF Residual	*Power in the high frequency band [0.2-1.5] for residual component*	0,008136	0,007163	0,004194	0,003463	0,002812	0,001931
LF/HF Residual	*Ratio of low-to-high frequency power for residual component*	9,541607	5,277584	11,3398	5,356435	9,448729	7,162101

Concerning the analysis of the two respiratory-related components of HRV frequency domain features, the HF RespComp showed an increasing trend during the protocol (Friedman's $\chi^2(2) = 7$, p-value = 0.030). However, this change was not statistically significant after correcting the p-values for multiple comparisons (pre vs. music V = 44, adjusted-p = 0.221; pre vs. post V = 52, adjusted p = 0.462; mus vs. post V = 84; adjusted-p = 1). No significant change has been reported for the LF/HF RespComp (Friedman's $\chi^2(2) = 2.1111$, p-value = 0.348).

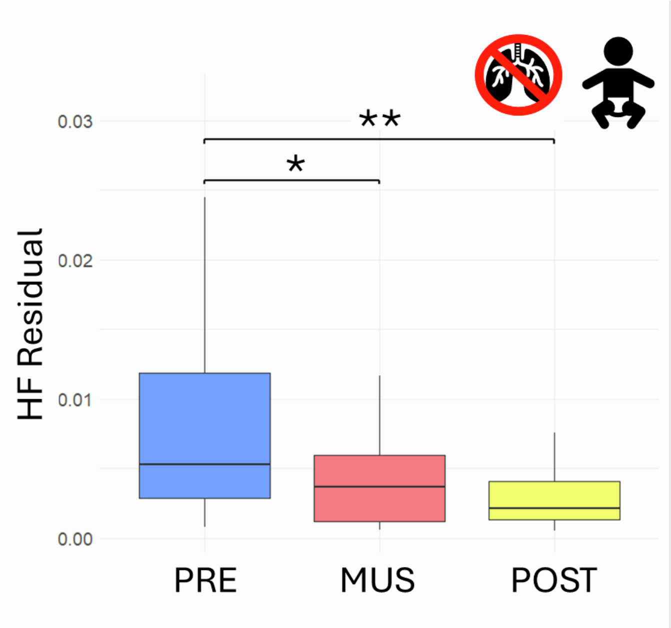

Figure S1. Boxplot representing the HF Residual component for preterm infants (n = 18) before (blue) during (red) and after (yellow) musical stimulation. Significance thresholds are represented by " = p <0.05", "** = p < 0.01". (Related to Figure 4).*

The results obtained for the HF Residual were consistent with the standard analysis, indicating a significant decreasing trend for HF (Friedman test, $\chi^2(2) = 12.333$, p-value = 0.002). Subsequent pairwise comparisons revealed a significant difference between the initial baseline and the music condition (V = 147, Bonferroni-corrected p-value = 0.017), and between the initial baseline and the post condition (V = 152, Bonferroni-corrected p-value = 0.007). No significant difference was reported between the music condition and the post condition (V = 126, Bonferroni-corrected p-value = 0.244). The trends of the values of HF Residual in the three sessions are presented in Figure s1.

No significant change has been reported for the LF/HF Residual (Friedman's $\chi^2(2) = 2.3333$, p-value = 0.311).

Table S2. Summary of metrics values obtained for adults before, during, and after the music protocol (Related to Table 2).

Adults

Metric	Description	pre		music		post	
		mean	std	mean	std	mean	std
HF RespComp	*Power in the high frequency band [0.15-0.4] for respiratory component*	0,347544	0,420832	0,158493	0,189496	0,189445	0,209955
LF/HF RespComp	*Ratio of low-to-high frequency power for respiratory components*	0,170688	0,215588	0,526094	0,740454	0,633318	0,924597
HF Residual	*Power in the high frequency band [0.15-0.4] for residual components*	0,124294	0,204784	0,162507	0,26016	0,124692	0,143426
LF/HF Residual	*Ratio of low-to-high frequency power for residual component*	3,587406	3,586123	4,961364	4,959509	5,053481	4,897669

The respiratory-related components exhibited significant variations, particularly in HF RespComp (Friedman's test, $\chi^2(2) = 14$, p-value = $0.009e^{-1}$). Post-hoc analyses revealed that HF RespComp was significantly higher in the initial baseline compared to the music condition (V = 181, Bonferroni-corrected p-value = $0.380e^{-3}$), and to the post condition (V = 169, Bonferroni-corrected p = 0.005), indicating a significant reduction in respiratory-driven vagal modulation during music exposure.

Conversely, LF/HF RespComp (Friedman's test, $\chi^2(2) = 9.5789$, p-value = 0.008). Post-hoc analyses revealed that LF/HF RespComp was significantly higher in the initial baseline compared to the music condition (V = 22, Bonferroni-corrected p-value = 0.006), no significant difference was reported for the other comparisons (pre vs. post: V = 42, Bonferroni-corrected p-value = 0.097; mus vs. post: V = 94, Bonferroni-corrected p-value = 1)

In Figure s2 are presented the boxplots reporting the median values of HF RespComp (left panel) and LF/HF RespComp (right panel).

No significant differences were observed in HF Residual (Friedman test, $\chi^2(2) = 0.73684$, p-value = 0.691), and the LF/HF Residual (Friedman test, $\chi^2(2) = 1.3684$, p-value = 0.505) in the three conditions.

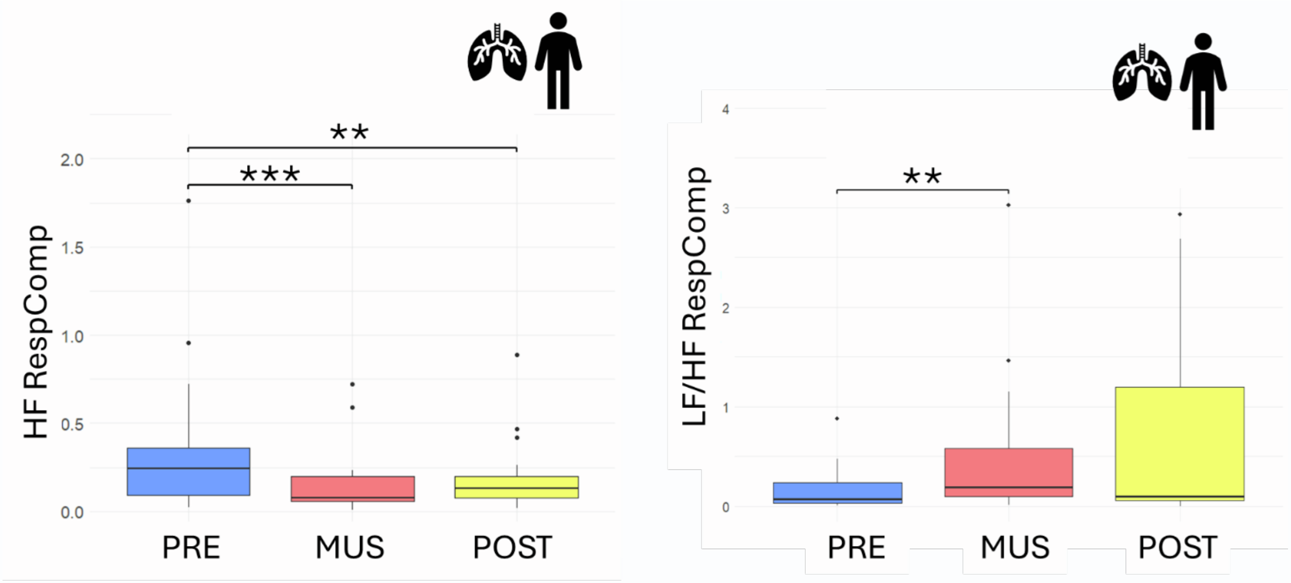

Figure S2. Boxplot representing the HF power(left) and LF/HF ratio (fight) of the respiratory component for adults (n = 19) before (blue) during (red) and after (yellow) musical stimulation. Significance thresholds are represented by " = p <0.05", "** = p < 0.01". (Related to Figure 5).*
